# Environmental Knights of the Roundtable: Stirring the Pot in Environmental Health

**DOI:** 10.1289/ehp.113-a28

**Published:** 2005-01

**Authors:** Ernie Hood

The Roundtable on Environmental Health Sciences, Research, and Medicine can be thought of as a think tank—a neutral, nonofficial setting where scientists, government officials, academics, industry representatives, and members of advocacy groups can gather to consider and discuss new scientific findings and emerging issues in environmental health. The roundtable, based at the Institute of Medicine (IOM) in Washington, D.C., is purposefully deliberative in nature. Its 34 members do not recommend specific actions or provide formal advice.

Despite that policy, the group has become highly influential in helping to shape the research agenda for fields in its purview. It has also helped what some observers felt was a disparate, fractured discipline to define itself more clearly and more broadly. By articulating a more holistic approach to environmental health, incorporating the natural, social, and built environments, the roundtable seeks to expand the dialogue, encouraging collaborations and partnerships among stakeholders. By identifying potential approaches to deal with new challenges, the group seeks to enhance the effectiveness of environmental health research and medicine in improving and protecting public health. “We try to find approaches toward making a contribution that would be unique to a body like the roundtable,” says NIEHS deputy director and roundtable member Samuel Wilson.

Roundtable workshops are designed to educate participants so that they can make informed decisions in their own arenas, whether they come from federal or state agencies, industry, or academia. The regional meetings are intended not only to provide information about regional environmental issues and concerns, but also to serve as outreach mechanisms, allowing local stakeholders to learn about new approaches while interacting with each other in the neutral roundtable setting.

Roundtable co-chair Lynn Goldman, a professor of environmental health sciences at the Johns Hopkins Bloomberg School of Public Health, finds the group’s many forms of interaction to be exciting and valuable. “Suddenly there’s an ‘a-ha’ in the room, where connections and collaborations are forming that weren’t there, and it’s out of that kind of stirring the pot that something like the roundtable can produce enormous benefits,” she says.

## Roundup in Houston

The roundtable has instituted a series of regional meetings across the nation. With its inherent neutrality, the roundtable seeks to facilitate dialogue among often-contentious groups in these regional meetings. “So often, these are individuals who only see each other in an adversarial setting,” says Goldman. “So it’s got to be healthy for people to be brought together in a setting where they can listen to each other, where they’re not directly at odds, where they’re not litigating each other, where they’re not fighting about what’s going to be in a regulation.”

Following on successful regional meetings in Pitts-burgh and Atlanta, the roundtable began 2004 with a January 23 conference in Houston. Like the two previous venues, the city and its surrounding region present a unique environmental situation, and a community atmosphere that the roundtable felt would be conducive to its message. “I often say Houston is ground zero for the interplay between many economic and environmental issues,” says Myron Harrison, senior health advisor for ExxonMobil Corporation and a roundtable member. “Houston has very real challenges. It’s a very business-oriented, high-growth, internationally oriented city. It won’t be able to achieve the growth and stature that is envisioned if it is difficult to attract new business and highly educated employees. Accomplishing this is in part dependent upon solving a broad set of air, land, and water issues.”

Goldman elaborates: “[Houston is] a place where there’s a very sharp interface between the human imprint on the land and the natural environment, a natural environment that’s fairly fragile. On top of that, there is a tremendous diversity in that community, with enormous issues of environmental empowerment, environmental justice, and social equality. All of that came to the fore in the workshop.”

Jane Laping, executive director of the local environmental group Mothers for Clean Air, was pleased that the roundtable chose to come to Houston. “It’s nice to finally be recognized,” she says. “We’re the fourth largest U.S. city, we’ve got the largest petrochemical complex here, we’ve got the worst ozone in the country for the fourth year now—it’s pretty bad. We really need some help.”

The one-day Houston workshop featured presentations on the many pressing environmental health issues in the region, including air pollution, water quality and flooding problems, urban sprawl, and obesity, as well as material on potential solutions such as sustainable growth, green buildings, and the importance of partnerships. “No one group, no one sector, no one set of stakeholders is going to solve anything by themselves,” says Harrison. “It only happens when you get these partnerships. . . . And we showed some good examples of partnerships at the Houston meeting.”

Wilson agrees that the Houston conference was successful in that regard. “There have been some significant follow-up activities that look very positive,” he says. “The scientists have become involved with the civic planners, also the environmental groups have made contact with the academics much more effectively as a result of the meeting. And it seems that the whole community down there is working on this topic in a much more tangible, enthusiastic, and robust way than it had before.”

The roundtable also held a regional meeting in Iowa in November 2004 (after this article’s press time) to examine the state of health and the environment in rural areas of the state.

## Emerging Issues: Nanotechnology

With the rapid development of nanotechnology, the roundtable felt the time was right to examine the potential environmental health issues involved with the expected proliferation of nanomaterials into virtually every aspect of commerce in the coming decades. By encouraging increased attention and research on the possible health and environmental pitfalls presented by nanomaterials, the roundtable hopes to contribute to the growing efforts among many stakeholders to maximize the enormous anticipated benefits of the technology by discovering and minimizing its associated risks.

The workshop, Technology and Environmental Health: Implications of Nanotechnology was convened in Washington, D.C., on 27 May 2004. Although many nanotechnology conferences have been held recently, Goldman says this one was different, thanks to the nature of the roundtable’s proceedings: “We were able to provide a neutral ground for discussion, and be able to hear from science leaders who are right at the cutting edge of doing toxicological assessments, leaders of industry who are right at the cutting edge of developing products, social science and policy experts who’ve been looking at the issue, and the people who are leading the nanotech efforts in the government.”

Presentations covered the gamut of issues related to nanotechnology, from potential applications in medicine and environmental remediation to potential health risks, along with discussions about societal implications and the importance of public perception to the technology’s ability to deliver on its promises. Ultimately, the meeting served as a forum for consideration of research needs in the area, to ensure that environmental health questions are answered before it’s too late to prevent negative impacts.

Says Christine Coussens, program officer of the IOM Board of Health Sciences Policy and study director of the roundtable: “The real purpose was to find out what’s missing in terms of a research agenda as the technology is developing.”

Goldman feels that the mix of attendees and the timing of the meeting were both fortuitous: “There’s a small group of experts in nanotechnology, and a number of them were on the agenda, but I think they had a different audience than they usually have, an audience that was very, very important in terms of bringing together the leadership from the federal health agencies. I was very pleased . . . that the meeting was able to be influential at a time that’s very critical.”

## A Meeting of the Minds on Disasters

On 22 June 2004, the roundtable held Public Health Risks of Disasters: Building Capacity to Respond, its first workshop in collaboration with another IOM group, the Disasters Roundtable. The conference was staged with the intent of integrating expertise and ideas from the two disciplines as well as increasing the role of public and environmental health considerations in disaster response. “The idea actually came from an internal request at the National Academies,” says Coussens. “The Disasters Roundtable hadn’t been spending a lot of their time talking about health and health risks associated with disasters. They knew a lot about disasters—infrastructure, communications, and other facets—and we knew a lot about health, and so we were asked to put together a workshop agenda that would look at some of the cross-cutting issues, trying to integrate between the two disciplines.”

Presenters at the meeting included emergency preparedness officials from the Department of Homeland Security and the Department of Health and Human Services, along with the director of the Centers for Disease Control and Prevention, public health and disasters experts from academia, and emergency management officials from both large and smaller metropolitan areas. With both natural and terrorism-related disasters seemingly inevitable, workshop participants stressed the need for enhanced collaboration and coordination among all those involved in disaster preparedness and response. They also advocated expanding preparation, mitigation, and response efforts to include hospitals, health care professionals, nongovernmental organizations, mass media, private businesses, academia, and the engineering and scientific communities. Many presentations explored the impacts of disasters on public health, including topics such as rapid assessment of health effects during disasters, infrastructure loss as a public health risk, and health effects of terrorism.

Roundtable member Jack Azar, who is vice president of environment, health, and safety at Xerox Corporation, was particularly anxious that the private sector be included in more multidisciplinary, integrated preparedness and response planning. “I was the only person from industry who spoke,” he says, “and what I asked was for industry to be included in the kinds of discussions that go on in emergency preparedness situations normally between government and nongovernment organizations [such as the Red Cross] only. One hundred million people are usually in the workplace when these things happen, so businesses really need to be brought into it as well.”

According to Goldman, the conference succeeded in increasing communication between the two fields. “I think this kind of meeting enriches the tools you have in your toolbox for doing things like assessing and managing risk, and hopefully helps to identify areas where more research, more information would be valuable for protection of the population. There’s more to it than screening your bags at the airport.”

## Spanning the Globe

With the next roundtable workshop, Global Environmental Health in the 21st Century: From Government Regulation to Corporate Social Responsibility, held 13–14 October 2004 in Washington, D.C., the group turned its attention to globalization as a potential driver of environmental health. Many companies in the United States are actually multinational, and they’re governed under a number of different countries’ regulations, says Coussens. One major question for the symposium was how this impacts environmental health in the United States and globally.

The initial thrust of the workshop was the concept of environmental management systems, the organized programs by which companies ensure adherence to high standards of environmental stewardship. For many companies, the concept is embodied in certification by the International Organization for Standardization under ISO 14001, an environmental management system that has been adopted around the world. But there is controversy over the effectiveness of such standardized approaches. “It’s not clear how environmental management systems impact or help to minimize the impact on environmental health,” says Azar. “There hasn’t really been any work trying to look scientifically at the impacts, what the benefits have been of [companies] getting ISO 14001–certified.”

Environmental regulation was another major theme. Several industry representatives pointed out the challenges brought about by the increasing global diversity of regulatory approaches. “Twenty years ago, the United States had [environmental regulation] to itself,” says Harrison. “It set the regulations, and then everyone copied them. That’s no longer the case. . . . These days, the leading edge of regulation is in the European Union [EU]. The EU is much more aggressive.”

Industry is increasingly seeing what could be characterized as a globalization of environmental regulation, as multinational companies respond to requirements imposed by different countries. “What’s happening is you’re getting different regulatory regimes created,” explains Harrison, “but for these companies that operate all around the world, you can’t be developing one product for one country and one product for another country—they have to do it all the same.” This will be a fertile area for future research, as scientists investigate the impact of such globalization on environmental health.

Many companies are now moving beyond regulatory compliance to embrace a concept called corporate social responsibility, and the potential impact of that trend was very much on the workshop’s agenda. Today, says Harrison, companies are faced not just with health and safety expectations, nor just with environmental expectations, but also with a long list of what are called social indicators, which include biodiversity, fair labor practices, and human rights. “The corporate social responsibility agenda is driving every bit as much activity—and maybe more in some places and some companies—than is regulation,” he says.

Azar concurs: “Everybody understands we’ve got to go well beyond compliance to a more sustainable concept. But it can be defined in many different ways. It’s going to take a different form whether you’re a chemical company, an electronics company, an appliance company, or an automobile company.” The workshop included presentations from both advocates and skeptics of voluntary corporate responsibility measures. The workshop, says Coussens, also raised awareness of the transparency that is needed to ensure corporate social responsibility. “It’s got to be more than some kind of marketing gimmick,” she says. “It has to provide real, useful data.”

Wilson anticipates this workshop will have long-term ramifications for the field of environmental health. “Integrating industrial practices into environmental health more so that there’s a much better communication pathway between the two groups is very important, and I think there’s a lot of downstream work to do on that,” he says.

The roundtable had an ambitious agenda in 2004, and the future looks to be equally challenging. As the scope of the roundtable expands, it is likely that its influence upon the field of environmental health will continue to grow, as will its success in providing a unique forum for consideration of the many complicated issues it faces.

## Figures and Tables

**Figure:**
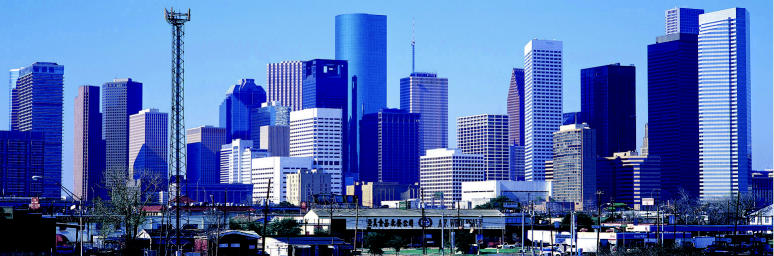
**Texas town meeting.** The roundtable met in Houston to examine the region’s environmental health issues.

**Figure:**
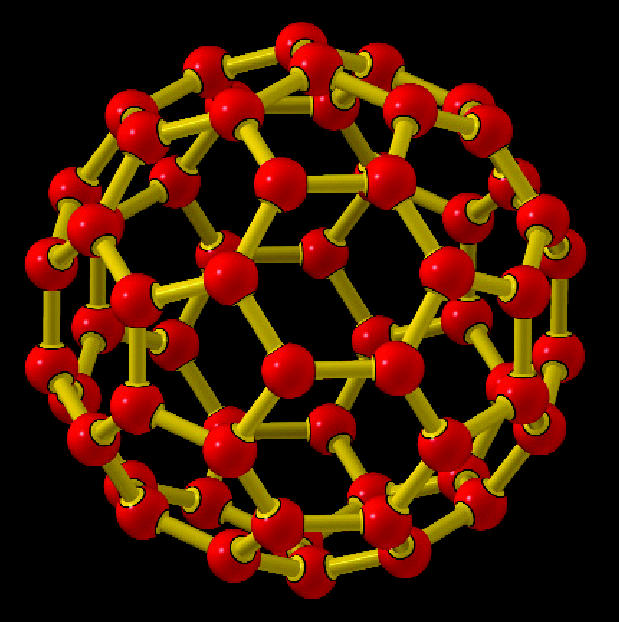
**Balancing buckyballs.** A workshop on nanotechnology looked at both the potential benefits and the potential dangers to health.

**Figure:**
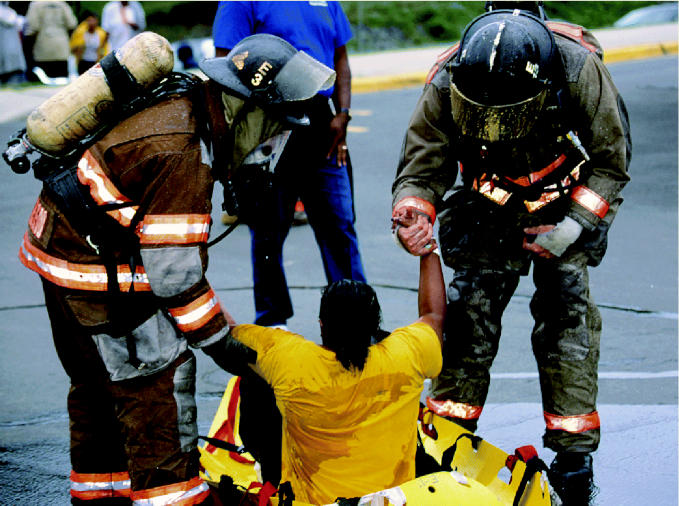
**Chance favors the prepared.** A workshop on disasters brought new perspectives to the thinking on preparedness and response planning.

**Figure:**
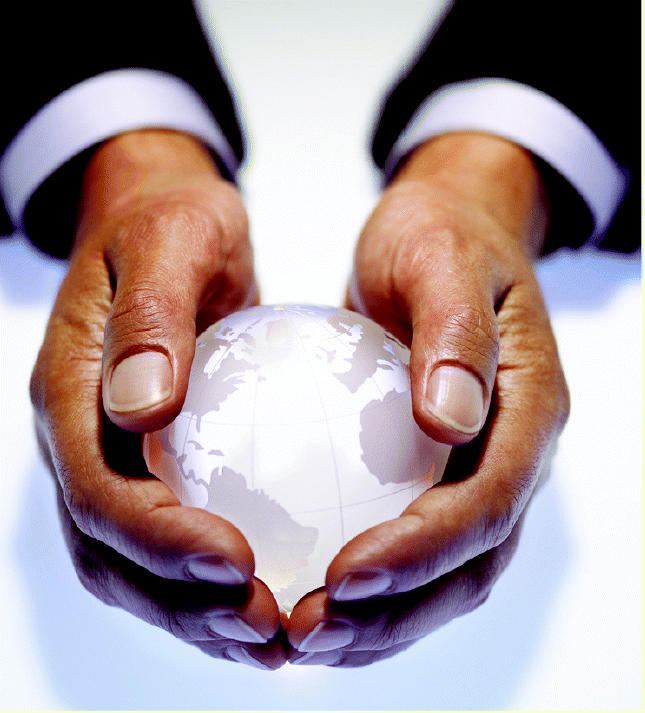
**Industrial evolution.** The move from government regulation to corporate social responsibility was debated at a recent roundtable workshop.

